# Can Flip-Chart Assisted Maternal Education Improve Essential New Born Care Knowledge and Skills? A Randomized Controlled Trial

**DOI:** 10.1007/s10995-022-03409-2

**Published:** 2022-04-06

**Authors:** Swathi Eluri, B. Shantharam Baliga, Suchetha S. Rao, V. Vinayagamoorthy, Nutan Kamath

**Affiliations:** 1 Department of Pediatrics, Kasturba Medical College, Mangalore, Manipal Academy of Higher Education, Manipal, Karnataka 575001 India; 2 Department of Community Medicine, Sri ManakulaVinayagar Medical College, Puducherry, India

**Keywords:** Breastfeeding, Knowledge retention, Maternal education, Neonatal mortality

## Abstract

**Background:**

Despite the implementation of essential newborn care (ENC) by the World Health Organization, knowledge gaps among postpartum women persist. Inappropriate breastfeeding practices and lack of knowledge regarding ENC among mothers has resulted in higher neonatal mortality.

**Purpose:**

Our study focused on evaluating the effectiveness of flip-chart assisted postpartum maternal education in improving ENC knowledge and skills.

**Material and Methods:**

A single blind parallel randomized controlled trial was carried out with 120 primigravidae. Participants were allocated to the intervention group (IG) or the control group (CG) by block randomization. A pretested validated questionnaire was administered to participants in both groups within 24 h post-delivery. Women in the IG were provided flip-chart assisted education regarding ENC approximately 24 h post-delivery. Women in both groups received verbal advice on ENC from the postnatal ward nurses, as per the existing hospital policy. ENC skills were observed in all participants in postnatal wards by independent observers. 6 months later, knowledge retention was assessed and analyzed in both groups.

**Results:**

Antenatal education remained at 32% among all postnatal women. Postnatal flip-chart-assisted maternal education had a significant impact on ENC skills in the IG (p < 0.01) and precipitated higher knowledge scores at the end of 6 months (p < 0.01) in the IG.

**Conclusion for Practice:**

Flip-chart assisted education soon after delivery had a sustained effect on ENC knowledge and practices that persisted for 6 months post-delivery.

## Significance

*What is already known?* Maternal knowledge has a significant role in improving neonatal health. Despite good antenatal coverage, a knowledge gap remains among mothers regarding essential newborn care.

*What this study adds?* Education with visual aids, like flip-chart assisted maternal education, provided soon after delivery had a sustained effect on essential newborn care knowledge and practices that persisted for 6 months post-delivery.

## Introduction

Ending all preventable newborn deaths and reducing neonatal mortality to as low as 12 per 1000 live births by 2030 is the target of the United Nations’ third Sustainable Development Goal (United nations, 2018). The World Health Organization (WHO) devised a comprehensive strategy of essential newborn care (ENC) intending to improve the health of newborns through interventions before conception and during the perinatal period (Narayanan et al., 2004). Although WHO has implemented ENC as of 2004, contrary to objectives of the program, inappropriate breastfeeding practices, malnutrition, and lack of knowledge regarding newborn care among mothers has resulted in higher neonatal mortality (Black et al., 2013; Chandhiok et al., 2015; Dongre et al., 2009) (Indian Institute for Population Sciences (IIPS) 2017; Patel et al., 2010; UNICEF, 2015). In India, exclusive breastfeeding (EBF) was practiced by only 48.6% of lactating mothers as per National family health survey 3(NFHS-3) as compared to 46.3% in National family health survey 1(NFHS-1) (Chandhiok et al., 2015). In their 2013 systematic review, Haroon et al. concluded that maternal education, either in groups or individually, is an effective means of increasing EBF practice (Haroon et al., 2013).

Knowledge gaps in ENC and deficiencies in skill domains are being observed among health care providers at various levels of the health care system (Malhotra et al., 2014; Pemo et al., 2019). Good antenatal coverage does not necessarily translate into good intranatal/postnatal practices among mothers (Gul et al., 2014). We aimed to assess knowledge of ENC at the time of admission for delivery and study the effectiveness of visual aid like flip chart assisted education soon after delivery in improving ENC knowledge and skills.

## Methods

### Settings, and Participants

This single-blind parallel randomized controlled trial was conducted at the Government Lady Goshen Hospital (LGH) and the Regional Advanced Paediatric Care Centre (RAPCC), Mangalore, in the district of Dakshina Kannada of coastal South India. These community hospitals are referral centers for tertiary care and are affiliated with Kasturba Medical College, Mangalore, Manipal Academy of Higher Education, Manipal. The study was conducted from January 2016 to September 2017.

Primigravida women (women who became pregnant for the first time) who vaginally delivered full-term babies weighing 2500 gm or more were included in the immediate postpartum period after informed consent. These women were healthy and could successfully breastfeed their babies. We included primigravida mothers as they do not have prior experience of newborn care. Multipara are excluded since they have cared for newborn babies and uniformity of subjects would be lost. Women with antenatal or postnatal complications were excluded from the study as they may not be able to express their ENC knowledge or take part in flip chart assisted educational intervention in the early neonatal period. Women whose neonates required neonatal intensive care (NICU) were also excluded from the study as these babies may not receive exclusive breastfeeding and the mothers may not be perceptive to knowledge about ENC care when their neonate is sick and in neonatal intensive care unit. The study participants were 120 primigravida women who met the inclusion and exclusion criteria. Using the software Open Epi version 3.0, the necessary sample size was calculated to be 120 (60 per group), based on a previous study, (Mazumder et al., 2014) with a 95% confidence interval, 80% power, and 10% attrition.

### Procedure

This study was approved by the Institutional Ethics Committee of Kasturba Medical College Mangalore (IEC KMC MLR 01-16/02) and was enrolled in the Clinical Trial Registry of India (CTRI/2016/04/006840).

Block randomization was carried out to assign study participants into two groups, intervention (IG) and control (CG). Random allocation software version 1.0 was used by an independent person to generate the random sequence with an equal block size of six and 12 total blocks. Allocation concealment was done by handing over the sealed, opaque, sequentially numbered envelope containing the random sequence and explaining the execution procedure to the resident doctor involved. Socio-demographic details were recorded and a pretested questionnaire was distributed among mothers in both groups within 24 h of delivery. The questionnaire was designed based on ENC as prescribed by WHO to assess the knowledge domain regarding ENC after antenatal care and education by health care providers in the field (WHO, 2002).

### Intervention

Flip-chart assisted maternal education was provided to the women in the IG after the questionnaires were collected. A flip chart was designed to communicate ENC knowledge and skills. The flip charts demonstrated breastfeeding techniques, newborn care, hygiene practices, and danger signs. Material for the flipcharts were taken from the WHO manual. On ENC (WHO, 2002). The flip charts were approved by the pediatric department and the institutional scientific research committee of Kasturba Medical College, Mangalore. Study tools were prepared in English and then translated into the local language of Kannada. The reliability of the questionnaire was tested using Cronbach’s alpha (0.752). The intervention of flip chart assisted ENC education was carried out by the principal investigator on the second postnatal day. Mothers were explained about the ENC, in their vernacular language with the help of flip charts in small group sessions of 2–3 mothers for 15–20 min duration. Participants in the CG and IG received verbal advice on ENC from the postnatal ward nurses, as per the existing hospital policy.

### Outcome Measures

On postnatal day two, participants in both groups were independently observed by trained nurses or resident doctors to assess their breastfeeding skills, thermoregulation, and hand hygiene practices. Observers were trained by the principal investigator and were blinded to study groups. These skill practices were documented separately by each observer and entered in a provided observation catalog. Routine postnatal care and care continuum were maintained in both groups, as per public health directives from the health department.

At the end of 6 months, women in both groups answered the same questionnaire to assess retention of knowledge and skills practiced.

### Data Analysis

Data were analyzed using IBM SPSS for Windows, version 25.0 (IBM, Corp., Armonk, NY). Each item on the questionnaire was allotted a particular score, and the median scores were compared using the Mann − Whitney U test and Wilcoxon signed-rank test. Correlation coefficients were calculated using Spearman’s correlation. Mothers’ knowledge was compared using a chi-squared test. A p-value < 0.05 was considered significant.

## Results

### Participants’ Baseline Knowledge and Practices

**Fig. 1:**
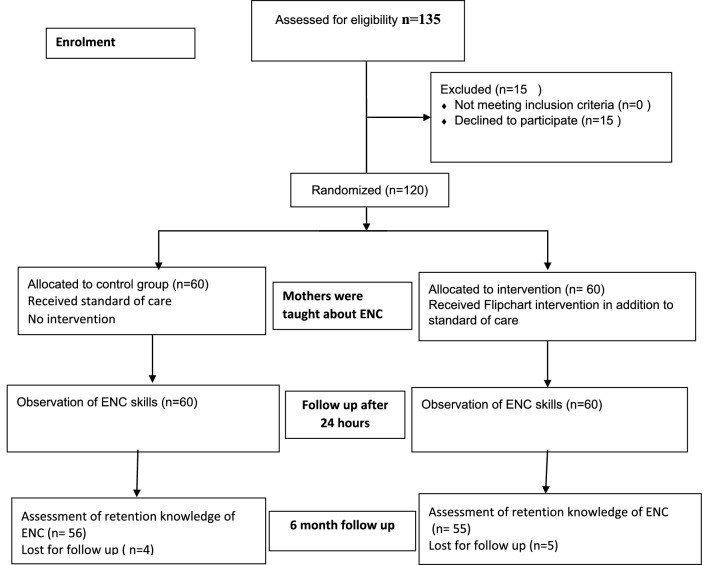
The flow diagram of the participants through the study. *ENC* Essential newborn care

A total of 120 postpartum women were enrolled in the study. They were grouped into either the CG or IG, each with 60 participants, by block randomization (Fig. 1). Both groups had similar demographic characteristics. Out of the 120 mothers in the study, 68.3% had received neither group nor individual education at any time during the antenatal period. Early initiation of breastfeeding (within 1 h) was practiced by only 48.3% and 53.43% of mothers in the CG and IG respectively, despite delivering at the facility.

Prelacteal feeds were provided by 6.7% of mothers in the CG and 15% of mothers in the IG. There was no statistically significant difference in the baseline ENC knowledge and practices between groups, as shown in Table 1.

**Table 1:** Comparison of baseline knowledge and practices about essential newborn care between control and intervention groups

Parameter	CG (n = 60) N (%)	IG (n = 60) N (%)	p-value
Colostrum should be given to the baby			
Agree	58 (96.7)	60 (100)	
Disagree	2 (3.3)	0	0.15
Intended duration of exclusive breastfeeding			
3 months	1 (1.7)	1 (1.7)	
4 months	2 (3.3)	4 (6.7)	
5 months	3 (5)	2 (3.3)	
6 months	18 (30)	20 (33.3)	0.9
> 6 months	11 (18.3)	14 (23.3)	
any other	11 (18.3)	8 (13.3)	
Do not know	14 (23.3)	11 (18.3)	
Technique of breastfeeding was shown antenatal			
Yes	36 (60)	38 (63.3)	
No	24 (40)	22 (36.7)	0.7
Technique of breastfeeding was shown by			
Doctor	3 (8.3)	4 (10.5)	
Nurse	10 (27.8)	13 (34.2)	
Mother	13 (36.1)	12 (31.6)	0.47
Family elders	10 (27.8)	6 (15.8)	
Health worker	0	3 (7.9)	
Extra diet necessary during lactation			
Agree	19 (31.7)	20 (33.3)	
Disagree	41 (68.3)	40 (66.7)	0.84
Methods to keep baby warm^*^			
Keeping in a warm room	4 (6.7)	1 (1.7)	
Wrapping with a cloth	47 (78.3)	43 (71.7)	
Covering the head with a cap	21 (35)	7 (11.7)	
Putting socks and gloves	6 (10)	2 (3.3)	0.07
Any other	2 (3.3)	4 (6.7)	
Do not know	10 (16.7)	14 (23.3)	
Handwashing necessary before handling baby			
Agree	27 (45)	26 (43)	
Disagree	33 (55)	34 (57)	0.24
Frequency of hand wash before handling the baby			
Once a day	3 (5)	1 (1.7)	
Twice a day	15 (25)	7 (11.7)	
Thrice a day	19 (31.6)	21 (35.0)	
Every time	10 (16.7)	17 (28.3)	0.30
Any other	12 (20)	13 (21.7)	
Never	1 (1.7)	1 (1.7)	
Danger signs to say a baby is not well ^*^			
Poor suck	0	4 (6.7)	
Poor activity	3 (5)	7 (11.7)	
Jaundice	1(1.7)	1 (1.7)	
Fever	7 (11.6)	13 (21.7)	
Hypothermia	0	0	0.16
Breathing difficulty	3 (5)	1 (1.7)	
Convulsions	1 (1.7)	0	
Any other	4 (6.7)	2 (3.3)	
Do not know	47 (78.3)	41 (68.3)	

p < o. o5 is statistically significant

*CG* control group, *IG* intervention group

*Multiple responses allowed

### Primary Outcomes

#### Outcomes in ENC Skills

In the IG, 60 women received ENC education. Breastfeeding skills, measures of thermoregulation and hygiene practices were demonstrated individually, in-person, and using flip charts. Significant increases in proper breastfeeding techniques, warm care skills, and good hygiene practices were observed among the mothers after education (Table 2).

**Table 2:** Comparison of essential newborn care practices of mothers post-intervention in the control and intervention group

Parameter	IG (n = 60) N (%)	CG(n = 60) N (%)	p-value
Breastfeeding in the proper position			
Yes	45 (81.8)	36 (64.3)	0.03
No	10 (18.2)	20 (35.7)	
Good latching			
Yes	46 (83.6)	32 (57.1)	0.002
No	9 (16.4)		
Breast engorgement/cracked nipple			
Yes	13 (23.6)	20 (35.7)	0.16
No	42 (76.4)	36 (64.3)	
Giving feeds other than breast milk			
Yes	13 (23.6)	27 (48.2)	0.007
No	42 (76.4)	29 (51.8)	
Wrapping the baby properly with a cloth			
Yes	50 (90.9)	45 (80.4)	0.11
No	5 (9.1)	11 (19.6)	
Putting a cap and socks			
Yes	40 (72.7)	14 (25)	< 0.001
No	15 (27.3)	42 (75)	
Washing hands before handling the baby			
Yes	46 (83.6)	25 (44.6)	< 0.001
No	9 (16.4)	31 (55.4)	
Washing hands before and after eating			
Yes	43 (78.2)	49 (87.5)	0.19
No	12 (21.8)	7 (12.5)	
Short and clean nails			
Yes	43 (78.2)	23 (41.1)	< 0.001
No	12 (21.8)	33 (58.9)	
Wearing the same dress for 2 days in a row			
Yes	20 (36.4)	39 (69.6)	< 0.001
No	35 (63.6)	17 (30.4)	

p value < 0.05 is statistically significant

*CG* control group, *IG* intervention group

### Outcomes in Knowledge Retention After 6 Months

At the end of 6 months, EBF, warm care practices, and maintaining hand hygiene was higher in the IG (Table 3). The ability to recognize danger signs in the baby was also significantly higher in the IG (Table 3). The incidence of illness among infants was significantly less in the IG; only 24.1% in the IG had febrile illness as opposed to 61.8% of the CG. The knowledge domain score was calculated as the median score based on the interquartile range. The post-intervention knowledge domain score at 6 months was higher in the IG (14, as opposed to 6 in the CG), suggesting the immediate postpartum period as ideal timing for education (Table 4).

**Table 3:** Comparison of essential newborn care knowledge 6 month following the intervention in control and intervention groups

Parameter	CG (n = 56) N (%)	IG (n = 55) N (%)	p-value
Colostrum should be given to the baby			
Agree	56 (100)	55 (100)	
Disagree	0	0	0.8
Duration of exclusive breastfeeding			
3 months	3 (5.4)	0	< 0.001
4 months	2 (3.6)	0	
5 months	5 (8.9)	1 (1.8)	
6 months	17 (30.4)	47 (85.5)	
> 6 months	14 (25)	5 (9.1)	
any other	12 (21.40	2 (3.6)	
Do not know	0	0	
Extra diet necessary for lactation			
Agree	16 (28.6)	52 (94.5)	< 0.001
Disagree	40 (71.4)	3 (5.5)	
Methods to keep baby warm^*^			
Keeping in a warm room	5 (8.9)	26 (47.3)	< 0.001
Wrapping with a cloth	49 (87.5)	54 (98.2)	
Covering the head with a cap	21 (37.5)	55 (100)	
Putting socks and gloves	8 (14.3)	42 (76.4)	
Any other	1 (1.8)	5 (9.1)	
Do not know	3 (5.3)	0	
Handwashing necessary before handling baby			
Agree	24 (42.8)	48 (87.2)	0.01
Disagree	32(57.2)	7 (12.8)	
Frequency of hand wash before handling the baby			
Once a day	2 (3.6)	0	< 0.001
Twice a day	18 (32.1)	0	
Thrice a day	17 (30.4)	9 (16.4)	
Every time	9 (16.1)	46 (83.6)	
Any other	10 (17.8)	0	
Never	0	0	
Danger signs to say a baby is not well ^*^			
Poor suck	0	37 (67.3)	< 0.001
Poor activity	5 (8.9)	50 (90.9)	
Jaundice	0	44 (80)	
Fever	9 (16.1)	52 (94.5)	
Hypothermia	0	19 (34.5)	
Breathing difficulty	2 (3.6)	50 (90.9)	
Convulsions	0	34 (61.8)	
Any other	2 (3.6)	0	
Do not know	43 (76.7)	0	

p < 0.05 is statistically significant

*CG* control group, *IG* intervention group

**Table 4:** Knowledge domain scores pre-intervention and 6 months post-intervention

Timeline	Median score (IQR)		p-value
	Intervention group	Control group	
Pre-intervention	7 (5.2–8.7)	7 (5–9)	0.40
6-month post intervention	14 (13–14)	6 (4–8)	< 0.001

p value < 0.05 is statistically significant

*IQR* Inter quartile range

## Discussion

Despite several programs and multipronged approaches, EBF for 6 months, and “good ENC practices” were not universal (Meshram et al., 2019). The present study was intended to assess whether providing education to women immediately after delivery had a better and longstanding impact on ENC knowledge and skills. In the study area, which has a female literacy rate of 84.13%, (Indian Population Census, 2020) only 32% of expectant mothers had received antenatal education despite 97% antenatal coverage. A low percentage of breastfeeding initiation within 1 h (48.3% in the CG, 53.4% in the IG) and the introduction of prelacteal feeds were indicative of the ineffectiveness of counseling during antenatal visits, with potentially serious consequences. Awasthi et al. found very low rates of EBF and a lack of proper knowledge on complementary feeding to be contributors to severe acute malnutrition in northern India (Awasthi et al., 2019). A study by Madhu et al. in South India reported practices such as discarding of colostrum and improper complementary feeding practices; the researchers emphasized the need for breastfeeding educational programs during antenatal and postnatal check-ups (Madhu et al., 2009). In the present study, low knowledge domains regarding ENC among all mothers pre-intervention indicated either ineffective antenatal education or poor receptiveness among expectant mothers. There was also lack of uniformity in the persons imparting the knowledge on ENC in the antenatal period in both groups. Their knowledge on ENC is variable and proportional to prior training and experience. Mothers and family elders providing the knowledge on newborn care may not cover all aspects of ENC. Their advice may be influenced by their cultural and religious customs (Pemo et al, 2019).

Improved breastfeeding practices, hygiene skills, and temperature maintenance after the educational intervention reflected enhanced receptiveness arising out of need. This significant behavioral change needs to be leveraged for better neonatal care. The loss of knowledge and skill domains after antenatal education is the consequence of a long interval between education and practical application in the postnatal period. In contrast, knowledge application and skill practices happened instantaneously after education in the IG in the present study, which resulted in higher scores after 6 months. Lower scores in the knowledge and skill domains in the CG despite the care continuum indicated the ineffectiveness of healthcare workers in caring for lactating mothers. Advice that was not reflective of the expectations and experiences of primiparous women caused anxiety among mothers and subsequent failure of lactation and EBF (Malouf et al., 2019).

The technique of breastfeeding was described verbally in the immediate postnatal period by similar personnel for both groups as per our hospital protocol. The number of subjects who were informed about the breast feeding technique was also similar in both groups. However with our intervention we demonstrated significantly higher EBF in the IG, compared with the CG at 6 months of age of their infants. The timing of intervention, the expertise and training of personnel imparting the education and the tools provided for communication contributed significantly to the mothers knowledge and skills on ENC.

A cluster randomized controlled trial in Ghana involved an intervention in which health care providers were trained to use the continuum of care cards as an educational tool to provide primary postnatal care to women, either in a health care facility or through home visits. The intervention translated into a better quality of care (Okawa et al., 2019). Higher rates of EBF were due to higher confidence levels and good dietary practices adopted post-intervention (Okawa et al., 2019).Strengthening efforts to enhance mothers’ self-confidence is essential in encouraging EBF, especially in countries with low EBF rates (Adhisivam et al., 2017; Chandhiok et al., 2015; Haroon et al., 2013; Opadeyi et al. 2019; Victora et al., 2016; Tsegaye et al., 2019). The present study established that the best breastfeeding practices with long term benefits can be achieved by reinforcing education on ENC soon after delivery.

Knowledge of danger signs in newborns considerably increases mothers’ health-seeking behavior, thereby increasing the chances of child survival. Maternal knowledge of danger signs in the IG was significantly higher 6 months post-intervention (Table 3). Thus, the present study established that the immediate postpartum period is the ideal time for maternal education to achieve good breastfeeding, thermal care, and hygiene practices, followed by EBF for 6 months, for overall improvement in neonate and infant health. The educational intervention followed by the practical demonstration and immediate practice on their own neonate had a significant impact compared to the routine verbal counselling by the ward nurses.

### Limitations

The place of intervention was urban. Studies involving childbirth in rural regions of India, may be required to generalize the applicability of this intervention. Under ordinary circumstances ENC education is provided by healthcare workers like ward nurses and lactation counsellors. They could be trained to give the flip-chart aided education under the intervention. We have not collected the information about expectations of primiparous women regarding ENC education. Further studies on impact of educating mothers with previous children, mothers with multiple pregnancies, and with neonates who required intensive care in the early neonatal period are recommended.

## Conclusion for Practice

Significant increases in proper breastfeeding techniques, warm care skills, and good hygiene practices were observed among mothers after receiving postnatal flip-chart assisted education. Furthermore, compared with the control group, the intervention group demonstrated an improved ability to recognize danger signs in newborns and had reduced rates of illness in early infancy. Maternal education soon after delivery had a sustained effect on ENC knowledge and practices that persisted for 6 months post-intervention. Mothers are perceptive to receive information about caring for their newborn immediately after childbirth. Demonstration of ENC skills by the health care provider and practice on their newborn under supervision helps in knowledge retention. Further irrespective of the number of antenatal visits, all mothers will have their delivery in a healthcare facility. The peripartum period is available to healthcare providers for educating mothers on ENC. Training these personnel on ENC will maintain uniformity of imparting knowledge and skills regarding ENC. Mothers will also have an opportunity to clarify their doubts or difficulties after practicing ENC on their neonates. Thus, education regarding ENC provided during this period is an effective intervention for lactating mothers to improve the care of their infants.

## Data Availability

Data can be made available on request.

## Appendix 1 (Questionnaire)

### Questionnaire to Compare the Baseline Knowledge About Essential Newborn Care Between Control and Intervention Groups

**Table:**

1	Did you receive education regarding new born care during your antenatal visits?
	Yes
	No
2	Do you feel colostrum should be given to the baby?
	Agree
	Disagree
3	When did you initiate breast feeding your baby ?
	Within half an hour
	Between half to 1 h
	Between one to 2 h
	Beyond 2 h
4	What did you feed the baby ?
	Direct breast feed
	Formula feeds
	Water
	Honey
	Both breast feed and formula feed
	Any other
5	How long you intend to exclusive breastfeed ?
	3 months
	4 months
	5 months
	6 months
	> 6 months
	Do not know
7	Were you shown the technique of breastfeeding during antenatal visits ?
	Yes
	No
8	If answer to question 7 is yes, who showed the technique of breastfeeding ?
	Doctor
	Nurse
	Mother
	Family elders
	Health worker
9	Do you feel extra diet necessary during lactation ?
	Agree
	Disagree
10	How will you keep your baby warm (choose whichever is applicable)?
	Keeping in a warm room
	Wrapping with a cloth
	Covering the head with a cap
	Putting socks and gloves
	Any other
	Do not know
11	Handwashing is necessary before handling baby
	Agree
	Disagree
12	How frequently do you wash hands before handling the baby?
	Once a day
	Twice a day
	Thrice a day
	Every time
	Never
	Any other
13	What are the danger signs to say baby is not well?
	Poor suck
	Poor activity
	Jaundice
	Fever
	Hypothermia
	Breathing difficulty
	Convulsions
	Any other
	Do not know

### Questionnaire to Compare of Essential Newborn Care Knowledge and Practices 6 month Following the Intervention

**Table:**

1	Do you feel colostrum should be given to the baby?
	Agree
	Disagree
2	How long did you exclusively breastfeed?
	3 months
	4 months
	5 months
	6 months
	> 6 months
	Do not know
3	Do you feel extra diet necessary during lactation?
	Agree
	Disagree
4	How will you keep your baby warm (choose whichever is applicable)?
	Keeping in a warm room
	Wrapping with a cloth
	Covering the head with a cap
	Putting socks and gloves
	Any other
	Do not know
5	Handwashing is necessary before handling baby
	Agree
	Disagree
6	How frequently do you wash hands before handling the baby?
	Once a day
	Twice a day
	Thrice a day
	Every time
	Never
	Any other
7	What are the danger signs to say baby is not well?
	Poor suck
	Poor activity
	Jaundice
	Fever
	Hypothermia
	Breathing difficulty
	Convulsions
	Any other
	Do not know
8	Did your infant got any febrile illness in past 6 months?
	Yes
	No
9	If answer to question 8 is yes, mention the number of episodes

## Care of Skin and Eyes

- To use warm water
- To test the water temperature before starting
- To use only clean water on the face and not to use soap
- To gently wipe each eye clean with the corner of a clean cloth, using a new corner for each eye, starting near the nose and wiping outward
- To dry each part thoroughly after washing it
- To separate the skin folds to look for any rashes
- To thoroughly dry inside the skin folds
- To look for signs of eye or umbilical infection
- To wrap the baby well

## Danger Signs

**Table:**

Poor suck Feeding difficulties
Poor activity
Jaundice
Fever
Hypothermia
Breathing difficulty
Convulsions
